# Jottings

**Published:** 1909-10-02

**Authors:** 


### JOTTINGS.

Hospital Saturday is fixed for October 16. The quarterly meeting of the board of delegates will be held at the offices of the Fund on Saturday, October 2, at 6 o'clock.

The Romford Guardians have accepted the terms of the Romford Joint Hospital Board to undertake the treatment in their hospital of pauper patients, at a charge of 35s. per week, but this sum is not to include cost of ambulance nor travelling expenses.

The Board of Guardians of Auckland have received the sanction of the Local Government Board to a loan of £6,250 for building a new infirmary.

The District Isolation Hospital Committee of Wincanton has accepted the tender of Mr. C. Bryer, jun., of Bridgwater, at £1,684, for the erection of new hospital buildings, together with roads, drains, fences, etc.

The Board of Guardians of Woolwich has accepted the tender of Messrs. F. and G. Foster, of Sunny Bank, Norwood Junction, for the erection of new open-air wards at a cost of £1,224.

The new Ramsgate General Hospital will be opened by Lord Brassey on Thursday, October 7, at 3 o'clock in the afternoon.

The Duchess of Albany, with Prince and Princess Alexander of Teck, will attend the entertainment given on behalf of the National Hospital for the Paralysed and Epileptic at 47 Brook Street, W., on October 8.

H.R.H. the Princess of Wales will receive the Jubilee Purses at the National Hospital for the Palalysed and Epileptic on Saturday, October 9, at 3.15.

Competitive designs are invited by the Urban District Council of Aberdare for a fever hospital for 52 patients, including administrative block, outbuildings, etc. Premiums, £100, £50, and £25. Apply, Mr. Thomas Phillips, clerk to the Council, Council Offices, Aberdare.

Extension to Cottage Hospital.—The tender of Messrs. Blay, of Dartford, for the extension to the Dartford Cottage Hospital has been accepted at £4,088.

Mrs. M. Brudenell-Murphy having generously contributed £100 towards the funds of the Royal Hospital for Incurables, Dublin, on September 14 was unanimously elected a life governor.

Miss Melita Brackenbury Pearson Keeling, of Glossop Road, Sheffield, who died on June 2, left £50 each to the Victoria Seamen's Home, Poplar, and the Jessop Hospital for Women, Sheffield.

Dr. Thomas Crawford Hayes, late Emeritus Professor of Medicine at King's College, left £100 to the King's College Hospital Building Fund.

The Lord Mayor of London, who was accompanied by Miss Truscott (representing the Lady Mayoress) and Sheriff Sir J. J. Baddeley, visited Plaistow in State on Saturday afternoon last to lay the foundation-stone of St. Mary's Hospital for Women and Children, which has been founded by the Rev. T. Given-Wilson.

It is proposed to utilise the columns of the old Manchester Infirmary and erect a clock tower on the site of the old institution. The lower part will be converted into a public shelter, which is much needed in Piccadilly.

Captain J. W. Garnier, who for nearly three years has been a member of the secretarial staff of the Dreadnought Hospital, Greenwich, has been appointed Secretary of the Hastings, St. Leonards, and East Sussex Hospital.

At a meeting of the subscribers to the Lady Clarke Memorial Fund in Bombay, it was decided that the amount realised, £1,566, should be handed over to the committee of the Convalescent Home for Children at Versova, a Bombay suburb. It will be remembered that Lady Clarke was the wife of Sir George S. Clarke, the Governor of Bombay.

---

Dr. John Milson Rhodes, a well-known authority on Poor-law administration, died suddenly on Saturday night at his home, Ivy Lodge, Didsbury, near Manchester. Dr. Rhodes was born at Broughton, Manchester, in 1847. He obtained his diploma in 1874, and after gaining the degree of M.D., Brussels, in 1879, opened a practice at Didsbury, where the remainder of his life was spent. He had been a member of the Chorlton Board of Guardians for thirty years, and was an overseer of the poor for the township of Didsbury. He was also the author of a pamphlet on workhouse dietaries, and helped to bring about many reforms in workhouse management. At the inquest held in Manchester on Monday, September 27, on Dr. Rhodes, the evidence showed that for two or three years he had suffered fro[illegible] serious heart trouble. On Sunday afternoon, having [illegible] amount of work to do, he gave himself a dose of 10 minir[illegible] of liquor strychnine. Later he seems to have feared that th[illegible] dose was too strong, for he consulted another medical ma[illegible] who gave him 2 drachms of potassium bromide. Abo[illegible] four hours after he had taken the strychnine he collapsed. The jury found a verdict of "Death from misadventure."

